# Climate Change and Air Pollution-Related Health Effects on Pain

**DOI:** 10.3390/ijerph22111721

**Published:** 2025-11-14

**Authors:** Pamela Kushner, Pranab Kalita, Frédérique Bariguian Revel, Christie Oliver, Mounika Nangineedi, Mary Cardosa

**Affiliations:** 1 Family Medicine, University of California Irvine Medical Center, Orange, CA 92868, USA; 2 Family Medicine, Long Beach Memorial Medical Center, Long Beach, CA 90806, USA; 3 OTC Global Category Medical Affairs, Haleon plc., Weybridge KT13 0NY, UK; pranbdr@gmail.com; 4 Global R&D OTC Category—Medical and Scientific Affairs, Haleon plc., 1260 Nyon, Switzerland; frederique.x.bariguian@haleon.com; 5 Public Policy and International Affairs (Corporate Affairs), Haleon plc., Weybridge KT13 0NY, UK; christie.x.oliver@haleon.com; 6 Global OTC R&D Pain, Haleon plc., Richmond, VA 23220, USA; mounika.x.nangineedi@haleon.com; 7 Department of Anaesthesiology, Hospital Canselor Tuanku Muhriz UKM, Jalan Yaacob Latif Kuala Lumpur, Bandar Tun Razak, Cheras, Kuala Lumpur 56000, Wilayah Persekutuan, Malaysia; mary.cardosa@gmail.com

**Keywords:** chronic pain, environmental pollutants, extreme weather, musculoskeletal pain, particulate matter, vulnerable populations

## Abstract

Climate change-related weather extremes and air pollution have wide-ranging health effects, with emerging evidence suggesting a potential influence on pain. This narrative review explores the relationship between climate-related weather parameters/air pollution with pain across various conditions, including chronic and acute musculoskeletal pain, postoperative pain, headache/migraine, dysmenorrhea, and chest pain. Included studies were published in 2014 or later. Findings indicate that higher humidity/dampness may exacerbate pain in individuals with knee osteoarthritis, while extremes in temperature and humidity are linked to a higher risk of gout arthritis attacks. No clinically meaningful associations were found between weather parameters and acute low-back pain. However, lower barometric pressure, elevated temperatures, and possibly higher humidity may influence postoperative pain levels. Headache and migraine episodes were more frequent during heat waves and periods of high humidity or rainfall, as well as in areas with elevated traffic-related air pollutants and particulate matter. Air pollution exposure was also associated with increased risk of dysmenorrhea, while lower temperatures and higher humidity correlated with more severe menstrual cramps. Temperature extremes were linked to chest pain in patients with asthma and other conditions. Overall, this review highlights the disproportionate pain-related burden of climate change and air pollution on women and emphasizes the need for further research.

## 1. Introduction

The accelerating pace of climate change and worsening air pollution pose profound challenges to global health. Rising global temperatures, more frequent extreme weather events, and elevated levels of fine particulate matter 2.5 (PM_2.5_) and gaseous pollutants have been firmly linked to respiratory and cardiovascular morbidity, reduced lung function, and premature mortality [1,2]. A key mechanism underlying these health challenges is prolonged inflammation and systemic stress, which heighten inflammatory responses and disrupt autonomic functions, causing persistent physiological and psychological strain [3,4,5].

Beyond their well-established impact on cardiopulmonary and mental health, environmental stressors may also influence pain, a dimension of public health that remains comparatively underexplored [6]. Pain is a multifactorial phenomenon shaped by biological, psychological, and social determinants. Climate-related variables such as temperature extremes, humidity, and atmospheric pressure may exacerbate pain not as isolated triggers but as integrated contributors acting through systemic pathways [7]. Mechanistically, environmental exposures may heighten inflammatory responses, disrupt autonomic regulation, and amplify mood disturbances, each of which can increase pain sensitivity, alter pain perception, and reduce tolerance [7]. Recent evidence supports these associations. Studies have reported that high humidity and damp conditions are linked to increased pain in knee osteoarthritis, while temperature and humidity extremes are associated with acute gout attacks [8,9]. Lower barometric pressure (BMP) and higher temperatures may influence postoperative pain [10]. Weather extremes, particularly heat waves, are associated with higher rates of headache and migraine, and air pollutants such as nitrogen dioxide and particulate matter are strongly correlated with increased migraine risk [11,12]. Similarly, exposure to elevated air pollution levels has been linked to dysmenorrhea, while low temperatures and high humidity exacerbate menstrual cramps [13]. Air pollution is also associated with psychiatric disorders, including anxiety and depression, which are well recognized to amplify pain perception and reduce coping capacity.

Despite emerging evidence, the relationship between climate-sensitive variables, air pollution, and pain remains fragmented and inconsistent across studies [14,15]. Methodological differences, reliance on self-reported pain outcomes, and geographic variability contribute to heterogeneous findings. These gaps highlight the need for a comprehensive synthesis of available evidence to better understand the intersection of environmental exposures and pain. This narrative review aims to address this gap by examining the associations between climate change-related weather patterns, air pollution, and pain outcomes across acute and chronic conditions. By integrating findings from diverse fields, the review underscores the importance of recognizing pain as a climate-sensitive health outcome. This perspective not only advances scientific understanding but also informs clinical practice and policy efforts aimed at mitigating the pain-related burden of environmental change.

## 2. Materials and Methods

MEDLINE and EMBASE were searched to identify studies on climate change and pain conditions, with separate MEDLINE searches performed to study the influence of weather-related parameters and air pollution on pain conditions.

Reference lists of included studies and existing reviews were checked for additional titles. Titles and abstracts identified by the initial search were limited to only those in English to facilitate assessment, with meeting abstracts and full-text articles published between 2014 and April 2024 to focus on recent evidence with current methodology, climate trends and its impact on health. This time frame was selected to capture contemporary evidence, align with current climate data, and reflect evolving scientific methodologies.

The following terms were used as free-text keywords, using Boolean operators and truncations to ensure comprehensive coverage: “climate change”, “global warming”, “weather”, “meteorological factors”, “air pollution”, “pain”, “chronic pain”, “migraine”, “arthritis”, “dysmenorrhea”, “musculoskeletal pain”, “chest pain” “air pollution”, “particulate matter”, “PM_2.5_”, “ozone”. Clinical studies, abstracts, reports, and review articles were included. Included studies were those that evaluated the association between weather parameters and/or air quality measures and pain intensity, frequency, or related symptoms. Studies were required to include at least 50 participants to reduce the risk of small-sample bias and enhance generalizability. Studies assessing severe or complex pain conditions such as pain associated with cancer, cardiac discomfort, trigeminal neuralgia, or sickle cell anemia, were excluded. Excluding severe pain conditions ensures a focused analysis of weather-related pain variations, minimizing confounding factors like medical treatments. Severe symptoms involve complex biological mechanisms and intensive interventions that may obscure weather’s direct influence. Limiting the scope to mild and moderate pain enhances applicability to the general population, providing clearer insights into natural pain fluctuations.

As a narrative review, this work did not follow a pre-registered systematic protocol; instead, articles were identified and prioritized by the author team based on relevance to the topic and subject-matter expertise, critically discussed among co-authors, and included in the synthesis only after consensus agreement.

## 3. Results

A total of 163 publications were identified from MEDLINE and EMBASE searches that examined association between various pain conditions and weather variables.

**Figure 1:**
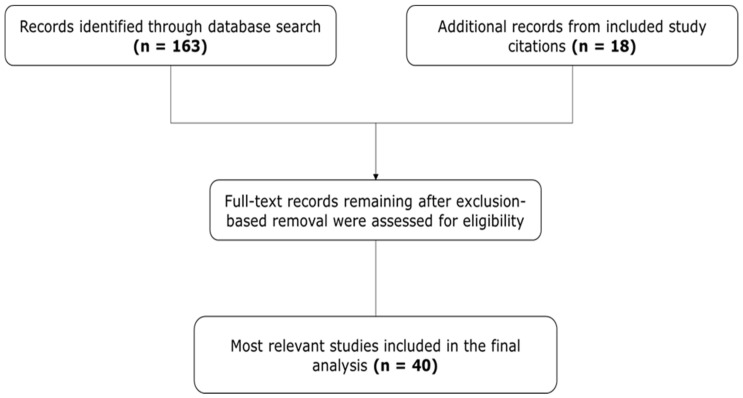
Flowchart Illustrating Study Selection Process.

**Figure 2:**
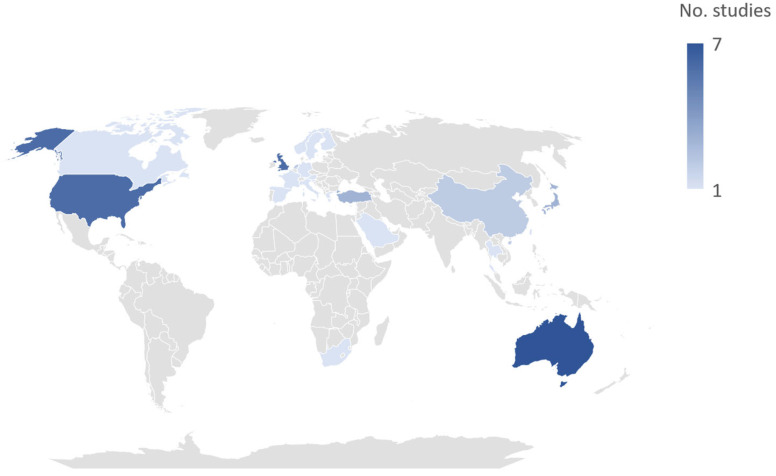
Geographical representation of the included studies (*n* = 40).

After screening and full text review, 22 articles met the predefined inclusion criteria. An additional 18 articles were identified from the separate Medline searches and by scanning the reference lists of included studies and review articles. Overall, the 40 most relevant studies were included (Figure 1). Figure 2 displays the number of studies included from each country. The included studies identified various pain conditions that appear to be influenced by meteorological parameters. The following sections examine the evidence for their susceptibility to weather and climate-related factors and explores possible mechanisms linking them.

The review included 40 studies. One study [16] included participants from 6 European countries (United Kingdom, Germany, Spain, Sweden, Italy, and Netherlands), and another study [17] included participants from 2 European countries (Greece and Slovenia). 

### 3.1. Chronic Pain (Multiple Conditions)

Four independent analyses of data from the UK-based smartphone app “Cloudy with a Chance of Pain” examined the relationship between weather parameters and pain in patients with chronic musculoskeletal conditions, neuropathic pain, or chronic headache, including migraine (Table 1) [18,19,20,21]. When participants rated the likelihood that weather affected their pain on a 0–10 scale, median response ranged from 7 to 8 [12,18,20,21]. Self-reported sensitivity was greatest dampness/rain, and humidity, although wind speed and BMP also contributed [18,19]. Sensitivity varied by conditions, people with fibromyalgia, chronic headache, and neuropathic pain were more frequently associate their pain with temperature changes and BMP [18].

Bayesian analysis revealed a modest relationship between pain and increased humidity, higher wind speed, lower temperature, and low BMP [21]. After adjustment for potential confounders (e.g., age, sex, activity), an estimated 1 in 10 patients were temperature-sensitive, 1 in 25 to relative humidity, 1 in 50 to BMP, and 3 in 100 to wind speed, with trends largely consistent across chronic pain conditions. Further analysis found that days with a high incidence of pain events were characterized by lower BMP, which was in turn associated with increased wind, moisture, and precipitation [20]. While these studies primarily focused on statistical associations and do not directly examine the physiological mechanisms underlying these relationships.

### 3.2. Chronic Musculoskeletal Pain

Studies highlight a link between weather, air pollution, and chronic musculoskeletal pain, summarized in Table 2. In a prospective time-series study of 94 patients with chronic rheumatic diseases, joint pain increased as temperature, relative humidity, and ozone (O_3_) decreased, and with higher BMP and nitrogen dioxide (NO_2_), however, no consistent association with PM_10_/PM_2.5_ was observed. In time-series analysis, temperature and NO_2_ remained independently associated with pain (10 °C fall in temperature ≈ 0.5-point increase on a 0–10 pain scale) [22]. Conversely, a retrospective analysis found no link between rainfall and joint or back pain-related outpatient visits [23]. Findings for osteoarthritis (OA) and weather sensitivity were mixed (Table 2). Data from the European Project on OSteoArthritis (EPOSA) showed 67.2% of participants reported weather-related effects on joint pain [16], most frequently for damp/rainy weather conditions (39.2%) and cold (30.2%), and less so for hot weather (4.6%). Women were significantly more likely to report weather sensitivity than men. In a cross-sectional study on knee OA, weather-sensitive patients had more severe knee pain, function, and structural abnormalities (cartilage defect and bone marrow lesions) after adjustment for age, gender, and body mass index [24]. Similarly, a case-crossover study among 744 adults with knee OA identified cold and damp weather as mild triggers for knee OA flares (odds ratio [OR] 1.45) [25].

BMP and humidity may affect postural stability and pain in patients with knee OA, although further study is needed to examine the directionality of possible interactions between postural stability and pain [26]. Two web-based crossover studies did not observe significant associations between weather parameters and joint pain in patients with knee [27] or hip OA [28], although one reported that greater temperature variation was linked to hip pain exacerbation [28]. Similarly, short-term exposure to PM_2.5_ was significantly associated with an increase in the number of outpatient visits for knee OA, and a strong exposure-response relationship was observed, with women and patients aged 65 years and older being more sensitive [29]. Among orthopedic implant recipients, 49% reported pain due to cold, though age and sex were not significant factor [30].

A study matching 14,200 patient-reported outcomes (PROs) to weather data in rheumatoid arthritis (RA) suggested slightly higher pain, fatigue, and disease activity scores during winter months, but correlations with meteorological variables were weak (Table 2) [30]. Similarly, an Austrian study highlighted that higher humidity, lower temperature, and lower saturation vapor pressure were significantly associated with greater pain among RA patients with moderate/high disease activity, while higher relative humidity, absolute humidity, vapor pressure or dew point had no effect on those with low disease activity and were associated with lower patient’s global assessment [31].

Similarly, an Austrian study found that higher humidity and lower temperature were associated with greater pain among RA patients with moderate–high disease activity, while such variables had little or even inverse associations in those with low disease activity [32].

For fibromyalgia, two studies examined the impact of weather parameters on pain in patients (Table 2). In one cohort of 50 women receiving Web-based counseling, pain decreased as BMP increased, but the strength of this relationship was not clinically meaningful; no significant associations were found with temperature, relative humidity, or solar flux [33,34]. A retrospective analysis likewise found weather-sensitive fibromyalgia patients reported higher pain and lower quality of life (QoL), but the magnitude of the association was not clinically meaningful [34]. In gout arthritis patients, higher temperatures and humidity extremes have been associated with greater risk gout attacks (likely mediated by dehydration and altered urate handling) (Table 2) [8].

**Table 2:** Relationship between weather parameters or air pollution and chronic musculoskeletal pain.

Study	Study Design and Participants	Key Findings
Multiple musculoskeletal conditions		
Jena et al., 2017 [23]	Retrospective US Medicare insurance claims database analysis linking rainfall data to outpatient visits for joint or back pain; included >1.5 million adults aged ≥ 65 years (62% women; mean [SD] age, 77 [0.3] years) and >11.6 million outpatient visits	No relation observed between rainfall and outpatient visits for joint or back pain–related problems including RA, OA, spondylosis, intervertebral disc disorders, and other nontraumatic joint disorders No differences between precipitation and outpatient visits for joint/back pain in subgroup analyses by age group, geographic region, race, or with vs. without RA
Ziade et al., 2021 [22]	Prospective, correlational analysis of data from 94 consecutive adult (≥18 years) pts (74.5% female; mean [SD] age, 56.4 [13.5] years) with chronic rheumatic disease (OA, RA, or SpA) consulting at 2 rheumatology clinics in Lebanon; pain assessed on NRS (rated 0–10) and correlated with air pollution parameters	82% of pts reported that weather variations affected their joint pain (cold, 66% of pts; humidity, 32%; heat, 14%), but only 18.1% had high pain variability over the year based on daily pain ratings Joint pain increased with decreasing temperature, relative humidity, and O_3_, and increased with increasing atmospheric pressure and NO_2_ (strength of associations was moderate); inconsistent correlations with particulate matters
OA		
Timmermans et al., 2014 [16]	Analysis of baseline data from the European Project on OSteoArthritis included 712 older pts with OA (72.0% female; mean [SD] age, 73.5 [5.5] years) from 6 European countries (Germany, Spain, Sweden, UK, Netherlands, Italy)	469 participants (67.2%) were weather sensitive ^a^ “Weather sensitive” pts reported significantly more joint pain vs. “non-weather-sensitive” pts In multivariate analysis, women were significantly more likely to report weather sensitivity than men Among weather-sensitive pts, 184 (39.2%) were sensitive to damp/rainy weather conditions, 145 (30.2%) were sensitive only to cold weather, and 23 (4.6%) were sensitive to hot weather Weather-sensitive people living in a warm/wet climate reported significantly higher pain intensity levels than those in a cold/wet climate (*p* < 0.001)
Ferreira et al., 2016 [9]	Web-based case-crossover study of 171 of 345 pts in Australia (64% female; mean [SD] age, 61.7 [8.7] years) with ≥1 knee OA exacerbation	No significant association between temperature, relative humidity, air pressure, or precipitation and risk of knee pain
Peultier et al., 2017 [26]	Prospective study of 113 pts in France with knee OA (69.0% female; mean [SD] age, 65.3 [9.2] years)	Significant relationships between weather parameters (atmospheric pressure, humidity), balance control, and pain Decrease in postural stability was observed when atmospheric pressure and maximum humidity decrease in the morning (*p* < 0.05) and when atmospheric pressure decrease within a day (*p* < 0.05) Increase in knee pain reported when it was warmer in the morning (*p* < 0.05) and then more humid and warmer within a day (*p* < 0.05)
Fu et al., 2020 [28]	Web-based case-crossover study of 129 pts aged ≥ 40 years (86% female; mean [SD] age, 62.9 [8.0] years) in Australia with hip OA and ≥1 episode of pain exacerbation	Greater variation in temperature over 3 days significantly associated with higher risk of hip pain exacerbation (*p* < 0.05 for linear trend) No significant association between maximum daily temperature, minimum daily temperature, relative humidity, precipitation, or BMP and hip pain exacerbations
Chen et al., 2021 [29]	Analysis of outpatient data from Beijing’s Medical Claims for Employees (urban employees) database from January 2010–December 2017 in conjunction with air pollution data; included 9,797,446 adult knee OA outpatient visits (pts: 63.6% female, 63.8% elderly)	After adjusting for confounding variables, every 10 µg/m^3^ rise in PM_2.5_ led to a 1.20% (95% CI, 1.20–1.21%) increase in the number of outpatient visits on the same day and a 1.41% (95% CI, 1.40–1.41%) increase in the number of outpatient visits when there was a lag of 0 to 3 days between the exposure and the visit The association between PM_2.5_ exposure and outpatient visits were slightly greater in pts aged ≥ 65 years vs. younger pts (1.59% [95% CI, 1.58–1.60%] vs. 1.28% [95% CI, 1.28–1.29%]) and in women vs. men (1.41% [95% CI, 1.40–1.41%] vs. 1.36% [95% CI, 1.35–1.37%])
Thomas et al., 2021 [25]	Web-based, case-crossover study in 744 adults (aged ≥ 40 years) in UK with knee OA (61% female; mean [SD] age, 62.1 [10.2] years) followed over 13 weeks; pts was asked, “Did the weather feel generally cold and damp?” during periods with and without OA pain flare	Generally cold/damp weather per pt report associated with increased risk of knee pain flare (OR, 1.45; 95% Cl, 1.12–1.87)
Xue et al., 2021 [24]	Cross-sectional study evaluating the association between self-reported weather sensitivity and clinical symptoms and structural abnormalities of the knee in a subsample of pts from a multicenter, prospective cohort study in China; analysis included 80 pts aged > 38 years with OA of the knee (75% women)	46 (57.5%) of pts reported weather sensitivity After adjusting for age, gender, and BMI, pts with weather sensitivity were significantly more likely to have severe knee pain (*p* < 0.05), severe knee dysfunction (*p* < 0.01), and severe overall clinical symptoms of the knee (*p* < 0.05), as well as severe cartilage defect (*p* < 0.05) and severe marrow abnormality (*p* < 0.05)
Rheumatoid arthritis		
Mandl et al., 2021 [31]	Daily correlation of clinical data from the Care for RA database with Austrian meteorological data over 12 years; included 461 patients (mean [SD] age, 55.3 [14.5] years)	Among patients with moderate/high disease activity, higher humidity was associated with greater pain, whereas higher temperature and higher saturation vapor pressure were associated with lower pain Among patients with low disease activity, meteorological parameters did not significantly correlate with pain, but higher humidity (relative and absolute), vapor pressure, and dew point were associated with lower PGA scores (indicating better overall health/lower disease activity)
Joly-Chevrier et al., 2023 [30]	Correlation of data from the RHUMADATA^TM^ clinical registry with Canadian meteorological data; included 14,200 weather-matched PROs (74.6% women)	Mean patient-reported pain, fatigue, and disease activity scores were higher in winter months among pts with RA, but the differences were small Disease activity was significantly higher in winter (*p* < 0.05) but was not significantly associated with any weather measures in winter or summer Few meteorological measures were significantly correlated with PROs, and all correlations were very weak
Fibromyalgia		
Smedslund et al., 2014 [33]	Analysis of data from the intervention group of a randomized, controlled trial of Web-based counseling, during which women in Norway with chronic widespread pain of FM recorded pain, activities, emotions, and thoughts 3 times/day over 5 weeks; data were correlated with meteorological variables; included 50 women (mean [SD] age, 43.0 [11.0] years)	BMP significantly associated with pain (*p* = 0.001), with pain increasing as BMP decreases, but relationship was too small to be clinically meaningful No significant associations between pain and temperature, relative humidity, or solar flux No significant interactions between psychological variables (e.g., emotions, psychological reactivity, and coping) and effects of weather parameters on pain
Hayashi et al., 2021 [34]	Retrospective analysis of data from medical records of consecutive patients aged > 20 years with FM during their first visit to a tertiary care center in Japan; correlated weather sensitivity ^b^ with pain intensity measured with an NRS (scores, 0–10) and QOL as assessed with the EQ-5D-3L scale; included 64 pts (75% female; mean [SD] age, 50 [16] years)	Pain severity values and QOL measures were statistically significantly (*p* ≤ 0.001) worse in pts with weather sensitivity compared with those without weather sensitivity The association with weather sensitivity and QOL was clinically meaningful The association with weather sensitivity and pain did not meet the threshold for clinical significance
Gout arthritis		
Neogi et al., 2014 [8]	Internet-based case-crossover survey of triggers of recurrent gout arthritis included 619 adults aged ≥ 18 years with a recent history of gout residing in the US (78% male; median [range] age, 54 [21–88] years)	Higher temperatures over the past 48 h were associated with an increased risk of recent gout attacks (*p* = 0.01 for linear trend); ~40% increase in risk of gout attack with higher vs. lower temperatures Extremes of humidity (low and high) were associated with higher risk of gout attack (*p* = 0.03 for quadratic trend)
Orthopedic implants		
Alakhras et al., 2020 [32]	Interview of 100 consecutive patients aged ≥ 14 years (56% male) with orthopedic implants followed up at an orthopedic department in Riyadh, KSA	49% of pts reported feeling pain related to cold conditions after implant surgery, and 29% reported significant pain No significant relationship between pain upon exposure to cold temperatures or pain severity and age or sex

^a^ Participants were considered as weather-sensitive persons when they indicated that damp/rainy, cold, and/or hot weather affected their joint pain. ^b^ Assessed with a single “Yes” or “No” answer to the question, “Does change in weather affect your pain?”. BMI, body mass index; BMP, barometric pressure; CI, confidence interval; EQ-5D-3L, Euro Quality of life-5 Dimensions-3 level; FM, fibromyalgia; US, United States; UK, United Kingdom; KSA, Kingdom of Saudi Arabia; NO_2_, nitrogen dioxide; NRS, numeric rating scale; O_3_, ozone; OA, osteoarthritis; OR, odds ratio; PGA, patient global assessment; PM_2.5_, particulate matter with particles with diameter < 2.5 μm; PRO, patient-reported outcome; pt(s), participant(s); QOL, quality of life; RA, rheumatoid arthritis; SD, standard deviation; SpA, spondyloarthritis.

### 3.3. Acute Musculoskeletal Pain

Three studies examined the relationship between weather parameters and acute low-back pain (LBP) in the primary care setting in Sydney, Australia [35,36,37]. A case-crossover study in 993 consecutive patients concluded that temperature, relative humidity, precipitation, BMP, and wind direction were not related to the onset of LBP [35]. In conditional logistic regression models, higher wind speed and wind gust produced small, but statistically significant increases in risk that were not considered clinically important. A prospective secondary analysis of 1604 patients from the PACE trial (short for “Pacing, graded Activity, and Cognitive behaviour therapy; a randomised Evaluation”) likewise found no association between weather variables (temperature, precipitation, humidity, and atmospheric pressure) and daily pain intensity across 14 days of follow-up [36]. Similarly, a case-crossover study (*n* = 981) in LBP patients found no significant association between most weather variables and the onset of acute LBP. Although one analysis found a small increase in odds with higher temperature and onset of LBP (OR, 1.20; 95% confidence interval [CI], 1.01 to 1.42; *p* = 0.03), a magnitude the authors considered unlikely to be clinically relevant [37].

### 3.4. Postoperative Pain

Weather parameters or seasonal effects may influence pain following surgical procedures. Shulman et al. analyzed 2369 outpatient visits from patients (55% female; mean age, 52 years) recovering after orthopedic surgery [10] and found that lower BMP was independently associated with increased pain over period of 1–60 months [10]. At the 1-year follow-up subset, lower BMP, higher temperature, and higher humidity were each significantly associated with increased pain [10]. In a separate Turkish study of 89 adults who underwent septoplasty or septorhinoplasty (52.8% male; mean [SD] age, 30.9 [10.9] years), postoperative pain scores were significantly lower at 1, 6, and 24 h in winter surgeries (all *p* < 0.05), than summer, but no difference was observed at 48 h [38]. Authors noted that summer’s dry, hot weather may worsen postoperative bleeding, but its effect on pain perception remains unclear.

### 3.5. Headache

Headache is among the most common neurologic conditions across all age groups and occurs more frequently in women than in men [39]. Table 3 summarizes studies examining the relationship between weather parameters, air pollution and headache or migraine. A retrospective observational study of 4375 individuals using a smartphone headache diary, accounting for confounding factor, found that lower BMP, BMP fluctuations, higher humidity, and more rainfall were significantly associated with more headache events [40].

Heat exposure during heat waves or hot-weather periods has been linked with headache across various populations (outdoor workers in Slovenia and Greece, individuals in South Africa, sugarcane workers in Thailand, and Australian miners) (Table 3) [17,41,42,43]. Thai sugarcane cutters working in the fields were at higher risk of heat stress than factory sugarcane workers and were significantly more likely to report headache ever (57.8% vs. 36.6%) and regularly (12.2% vs. 2.2%) [42]. In a retrospective cohort study of 18,065 adults with 22,021 emergency department (ED) visits for headache, higher ambient temperature was associated with a small but statistically significant increase in the relative risk (RR) of headache-related ED visits (RR 1.042 per 5 °C increase; 95% CI 1.009–1.076), with the effect most evident during the cold months. Higher levels of NO_2_ and PM_10_ were also associated with increased risk of headache-related ED visits, although effects varied by season. Headache ED visits associated with dust storms increased with age, and the risk associated with NO_2_ was highest among participants aged 40–60 years. These season- and pollutant-specific associations suggest that both meteorological conditions and episodic declines in air quality contribute modestly but significantly to ED presentations for headache [44].

A retrospective study among 3491 adults in Turkey found that higher temperatures and lower humidity were associated with higher numbers of ED visits for migraine, although no relationship was observed for BMP and wind speed [11]. A prospective diary-based cohort study of 98 adults with migraine that accounted for possible confounds (e.g., age, sex, socioeconomic status), found that higher relative humidity increased migraine risk. A 26.5% rise in the 3-day moving average was linked to a 28% higher likelihood of migraine onset, particularly in warmer seasons [45]. Temperature and BMP showed no significant effects, but in colder months, higher daily maximum 8-h O_3_ and carbon monoxide (CO) levels were weakly associated with an increased risk of migraine onset. A case-crossover study of 34,776 US patients with severe migraine (≥1 migraine-related inpatient or ED visit) found that a 25-mmHg increase in BMP increased the odds of severe migraine by 9% among patients taking prophylactic medication and by 56% among those not on prophylaxis [46]. In a cross-sectional, Web-based questionnaire study of 6786 adults with migraine, women identified weather changes as a migraine trigger significantly more frequently than men (45.9% vs. 38.7%) [47]. Similarly, a prospective study of 50 patients with episodic migraine and 50 with tension-type headache (TTH) reported that higher wind speed may be a risk factor for migraine attacks, whereas higher ultraviolet index may portend greater risk for TTH attacks [48].

**Table 3:** Relationship between weather parameters or air pollution and headache/migraine.

Study	Study Design and Participants	Key Findings
Vodonos et al., 2015 [44]	Retrospective cohort study correlating ED visits to a medical center in southern Israel with meteorological parameters and air pollutant levels in 18,065 patients aged ≥ 18 years (56.6% female, 59.9% < 40 years) with 22,021 ED visits for headache	Increase in temperature and NO_2_ was associated with increased risk of headache ED visits An increase in temperature by 5 °C was associated with a relative risk of ED visit for headache of 1.042 (95% CI 1.009–1.076; *p* = 0.005) Effect of the ambient temperature was most evident during fall (↑ 5 °C associated with RR 1.05; *p* < 0.05) and winter (increase 5 °C associated with RR 1.06; *p* < 0.05) Effect of NO_2_ was significant during winter (increase 10 units associated with RR 1.18; *p* = 0.001) and spring (increase in 10 units associated with RR 1.32; *p* = 0.001) Significant association between PM_10_ levels and ED visits on same day during fall season only Effect of temperature on ED visits did not increase with age Risk of headache ED visits associated with dust storms increase with age and associated with NO_2_ was highest in age group of 40–60 years
Yilmaz et al., 2015 [11]	Retrospective study correlating ED visits to a regional hospital in Turkey for migraine with meteorological data over a 12-month period; included 3491 patients (72% female; mean [SD] age, 36 [11] years)	Daily number of patients presenting with migraine was higher when daily maximum temperature was higher (*p* = 0.005), mean temperature was higher (*p* = 0.013), minimum temperature was higher (*p* = 0.041), daily temperature change was greater (*p* = 0.003), and daily average humidity was lower (*p* = 0.031) No significant correlations between daily number of patients presenting with migraine and BMP or wind speed
Pogačar et al., 2019 [17]	Cross-sectional study assessing perception of heat stress during heat waves among workers from Slovenia (*n* = 216, 57.4% male) and Greece (*n* = 70, 77.1% male) who spent > 1/3 of their workdays outdoors	52.8% of workers from Slovenia and 44.3% from Greece reported that they experienced heat-induced headache
Li et al., 2019 [45]	Prospective diary-based cohort study of 98 adults aged ≥ 18 years with migraine in the greater Boston, Massachusetts, area (88% female; mean [SD] age, 35 [12] years); total of 4406 days and 870 migraine headache episodes; fixed-effects models accounted for potential confounders (e.g., age, sex, socioeconomic status)	Higher relative humidity associated with increase odds of migraine headache onset (26.5% increase in 3-day moving average of relative humidity associated with a 28% [95% CI, 1.07–1.53] increase in odds of migraine headache onset); relationship significant in the warm season only No significant association of temperature or BMP with migraine headache onset Weak positive association observed for higher daily maximum 8-h O_3_ and daily maximum 8-h CO and increase odds of migraine headache onset in the cold season In sensitivity analyses accounting for potential correlations between temporally close migraine headache onsets within each pt, results for humidity and O_3_ were no longer significant, but associations by season remained
Wright et al., 2019 [41]	Cross-sectional survey about heat-related health effects experienced by individuals in Gauteng province, South Africa, during hot weather; included data from 136 households/580 individuals	Headache/nausea reported as a heat-related health effect in 19.1% of individuals
Boonruksa et al., 2020 [42]	Survey of 183 sugarcane workers (≥18 years of age) in Thailand who worked in the field (cutters, *n* = 90; 58.9% male; mean [SD] age, 42.0 [11.0] years) or in factories (*n* = 93; 94.6% male; mean [SD] age, 39.3 [7.5] years) during hottest month of harvesting; data on ambient temperatures and physiological stress also recorded	Sugarcane cutters were at higher risk of heat stress than factory workers Cutters were significantly more likely than factory workers to report headache ever (57.8% vs. 36.6%, *p* = 0.004) and regularly (12.2% vs. 2.2%, *p* = 0.008)
Lew et al., 2020 [46]	Case-crossover study using data from a database linking weather data with US administrative claims data; used conditional logistic regression to analyze the relationship between meteorological changes and severe migraines ^a^ in patients with and without prophylactic migraine treatment; included 34,776 patients with ≥ 1 severe migraine	After controlling for other weather conditions, each 25-mm Hg increase in daily maximum BPM was associated with a 9% increase in odds of severe migraine ^a^ (OR, 1.09; *p* = 0.025) among patients taking prophylactic medication and a 56% increase (OR, 1.56; *p* < 0.001) in patients not taking prophylactic medication
Akgün et al., 2021 [48]	Prospective analysis of weather parameters and frequency and severity of headache attacks recorded in diaries by 50 pts with episodic migraine (64% female; mean [SD] age, 37.0 [11.3] years; 188 headache attacks recorded) and 50 pts with episodic TTH (80% female; mean [SD] age, 34.4 [13.2] years; 233 headache attacks recorded); study in Turkey	Mean wind speed on days of migraine attacks was significantly higher than on days of TTH attacks (*p* < 0.05) Mean UV index was significantly higher on days of TTH attacks than on days of migraine attacks (*p* < 0.05) For TTH attacks, mean UV index (*p* < 0.05) and sunshine duration (*p* = 0.05) were higher in women, but no other significant sex differences for migraine or TTH attacks with regard to weather parameters Significant association between mean age of pts with migraine and mean air temperature on attack days (*p* < 0.05), whereby older pts had migraine attacks on colder days
Van Casteren 2021 [47]	Cross-sectional, Web-based questionnaire among 6786 Dutch adults with migraine (84.4% female; mean [SD] age, 41.9 [12.1] years in females, 45.7 [13.1] years in males)	Weather changes identified as a trigger for migraine attacks more frequently by women (45.9%) than men (38.7%; OR [95% CI], 1.35 [1.18–1.55], *p* < 0.001)
Katsuki et al., 2023 [40]	Retrospective, observational cross-sectional study in Japan; weather data and AI used to investigate the effects of weather parameters on headache occurrence; analysis included data from 4375 users of a smartphone app with an electronic diary who had moderate/severe headaches (89.2% female; mean [SD] age, 34.0 [11.2] years); 336,951 headache events; models accounted for variations in sex, age, date, and regional district	Lower BMP, changes in BMP, higher humidity, and more rainfall associated with increased number of headache events (all *p* < 0.001)
Taggart et al., 2024 [43]	Analysis of heat-related illness/injury data from a mine industry company in Australia over an 11-year period (March 2012–2023)	151 cases of heat-related illness due to outdoor exposure reported, with headache being among the most prevalent symptoms (23 cases) Significantly fewer injuries reported during cooler vs. hotter days (*p* < 0.001)

^a^ Defined as migraine-related inpatient hospitalization or emergency department visit. AI, artificial intelligence; BMP, barometric pressure; CI, confidence interval; CO, carbon monoxide; ED, emergency department; NO_2_, nitrogen dioxide; O_3_, ozone; OR, odds ratio; pt(s), participant; PM_10_, particulate matter with particles with diameter < 10 μm; RR, relative risk; SD, standard deviation; TTH, tension-type headache; UV, ultraviolet; ↑, Increase.

### 3.6. Dysmenorrhea

Lower temperatures, higher humidity, and elevated air pollutants may impact dysmenorrhea. In a nationwide longitudinal Japanese study (*n* = 319), lower temperature and higher humidity were significantly associated with increased diary-reported menstrual cramps after adjusting for possible confounding factors [49]. A US cross-sectional survey (*n* = 419; including 197 Asian participants) suggested that cold exposures (such as consumption of cold drinks/foods and cold home temperatures) may be associated with greater dysmenorrhea severity, particularly during winter months, although the relationships varied by race and geographic location [13].

In Taiwan, analysis of two national health insurance databases (296,078 women; mean [SD] age, 33.7 [10.4] years) found that among 12,514 women who developed dysmenorrhea over 12 years, exposure to the highest quartile of air pollutants was associated with significantly elevated risk of dysmenorrhea, with adjusted hazard ratios of 27.9 (nitric oxides; NO_x_), 16.7 (nitric oxide; NO), 33.1 (NO_2_), 28.7 (CO), and 27.6 (PM_2.5_) (*p* < 0.001 for each) after controlling for potential confounding factors. Moreover, the effects of air pollutants on dysmenorrhea risk appeared to be synergistic [50].

### 3.7. Chest Pain

Extreme high or low temperature may increase chest pain related to asthma and other conditions [51]. A population-based, cross-sectional study of 1995 adults with asthma in northern Finland (65.3% women, 58.2% aged ≥ 50 years) demonstrated a relationship between asthma severity/control and an increased prevalence of cold weather-related chest pain [52]. After adjusting for potential confounds, the highest prevalence ratios (PR [95% CIs]) were observed among women with severe uncontrolled asthma (4.48 [2.83–7.09]) relative to mild, controlled asthma and in men with severe, partly-controlled asthma (3.84 [1.72–8.57]) [50].

An Australian cohort study (*n* = 206,789) analyzed ambulance data from consecutive adult patients with nontraumatic chest pain (50.3% female; mean [SD] age, 61.2 [18.9] years). The results indicated a significant increase in both heat- and cold-related chest pain presentation at mean air temperatures above and below 20.8 °C. Discharge diagnoses ranged from nonspecific chest pain (48.1%) to cardiovascular (26.0%) and respiratory conditions (8.5%), among others [51]. Younger individuals and those of lower socioeconomic status appeared to be more susceptible to chest pain due to heat-related exposure, and individuals of lower socioeconomic status were also at higher risk of cold-related chest pain.

## 4. Discussion

### 4.1. Pain Variability Across Conditions

Climate change and air pollution are serious health concerns that contribute substantially to worldwide morbidity and mortality. Pain is an overlooked consequence of climate change and air pollution mainly due to its multifactorial etiology, the highly individualized nature of pain perception, and geographic differences. Evidence from multiple studies suggests that meteorological parameters play at least a partial role in the onset and severity of various pain conditions. Four analyses based on the UK-based “Cloudy with a Chance of Pain” study linked chronic pain conditions to lower BMP, higher humidity and precipitation, and stronger wind speed (Table 1). These findings suggests that atmospheric changes may influence pain perception, particularly in individuals with fibromyalgia, neuropathic pain, OA, and migraines. While geographical location clearly influences health outcomes, current literature remains inconclusive regarding which specific regions are more vulnerable to the pain-related effects of climate change. Although sensitivity to specific weather parameters varied, but the evidence supports the notion that a subset of patients experiences weather-related pain exacerbation. The mechanisms underlying these associations remain complex and not fully understood [34], but investigating them may offer insights into physiological pathways and inform targeted pain-management strategies. Also, this study [34] relied on single, one-time self-reports of pain or simple yes/no questions. While such measures offer ease of collection and straightforward analysis, they lack information on pain intensity, duration, and recurrence, are less sensitive to short-term change, and are more prone to measurement error; consequently, they weaken causal inference about the temporal relationship between environmental exposures and symptoms.

In OA, patients who self-identified as weather-sensitive reported more joint pain than non-weather-sensitive patients [16,24]. Physiological mechanisms have also been described, temperature and humidity shifts may affect tissue expansion, contraction, and synovial fluid viscosity, leading to increased stiffness and pain [16,24]. Additionally, research indicates that weather sensitivity plays a significant role in knee cartilage defects and bone marrow abnormalities, reinforcing the role of climatic factors contribute to joint degeneration. These findings collectively suggest that environmental changes may directly impact joint structures and pain perception in OA patients [16,24]. Also, higher humidity/dampness was associated with increased pain in patients with knee OA (Table 2) [25,26]. In RA, sensitivity to weather parameters may differ by disease severity. One study demonstrated increased pain in association with higher humidity, lower temperature, and lower saturation vapor pressure [31]. In gout, a single study documented significant associations between higher temperatures and extreme humidity (both low and high) with an increased risk of attacks by promoting dehydration and metabolic acidosis leading to reduced renal urate excretion and increased serum urate levels. Behavioral factors, like increased alcohol consumption in hot weather, may also contribute to this risk [8]. For acute LBP, studies generally did not demonstrate any clinically meaningful associations with weather parameters and pain [35,36,37]. For postoperative pain, one study linked low BMP, higher temperature, and higher humidity to increased pain [10], while another observed greater postoperative pain during warmer, drier summer months [38]. In dysmenorrhea, lower temperatures and higher humidity are associated with an increase in menstrual cramps [13,49]. Moreover, research has identified several biological mechanisms by which cold exposure may worsen dysmenorrhea through vasoconstriction, heightened inflammation, hormonal dysregulation, and amplified pain perception driven by prostaglandin activity and microvascular disturbances [13]. Extremes temperatures have also been associated with chest pain in patients with asthma, nonspecific chest pain, or cardiovascular disease, contributing to higher rates of ED visits [51,52]. However, mechanisms for temperature-related increases in chest pain presentations are broad and disease-specific. Among patients with headache or migraine, several studies reported positive association with humidity, rainfall, and air pollution (Table 3). Reports from five countries link extreme heat to increased risk of headaches, particularly among outdoor workers. For example, sugarcane laborers exposed to intense heat experience higher rates of heat stress and headaches than their indoor counterparts [17,41,42]. Additionally, higher temperature may also be associated with an increased risk of ED visits for headache or migraine [11,44]. ED data provide useful population-level signals but are subject to multiple non-environmental influences including healthcare-seeking behavior, service availability, coding practices, seasonal infectious disease activity, and socioeconomic factors that may confound associations with weather or air pollution. We therefore interpret these findings cautiously and recommend future research employ repeated, validated pain measures, linkage to clinical records where possible, and analytic strategies (e.g., time-series methods with control for healthcare utilization, stratified analyses, or causal models) to better control for measurement limitations and confounding. These findings highlight the heightened risks posed by extremes heat events under climate change, especially among outdoor workers in countries with hot climates. The mechanisms linking heatwaves and high temperatures causing headaches and migraines are multifaceted and probably involve a complex interplay of environmental factors, neurobiological and physiological responses. The transient receptor potential (TRP) channels, particularly TRPV1 (activated above 43 °C) and TRPV4 (responding to 24–35 °C), play a key role in migraine development by triggering inflammation and releasing pro-inflammatory agents like calcitonin gene-related peptide (CGRP) and substance *p* [53,54]. Similarly, heat is also known to cause vasodilation, which may contribute to vascular migraine. In addition, dehydration from excessive sweating can disrupt homeostasis, shrink brain tissue, and stretch dural venous structures, provoking secondary headaches including migraine [55,56,57]. Direct exposure to prolonged sunlight and ultraviolet radiation may further influences CGRP and NO release in skin nerve fibers, promoting vasodilation and migraine onset [58].

Furthermore, it is projected that by 2050, global temperature may rise by approximately 3 °C which could further influence pain sensitivity [59]. One study has predicted an increase in temperature-related chest pain, especially during heatwaves [51]. Additionally, existing literature indicates that rising temperatures may exacerbate symptoms of headache and migraine, underscoring the potential implications of future climate change for pain-related health outcome [17,41,42,43].

### 4.2. Exacerbating Conditions

Climate change can exacerbate pain by altering environmental factors such as air pollution and by interacting with physiological vulnerabilities, age, and socioeconomic status. Elucidating these mechanisms is essential for designing effective health risk mitigation strategies.

#### 4.2.1. Air Pollution

Five studies evaluated the relationship between air pollutants, including PM, traffic-related (O_3_, NO_2_, and CO) and industrial gases, and pain outcomes in patients with chronic rheumatic disease [22], knee OA [29], headache [44], and dysmenorrhea [50]. In chronic rheumatic disease, pain was found to increase with lower O_3_ levels and higher NO_2_ concentrations, while PM levels showed no consistent association [22]. Conversely, in knee OA, increasing PM_2.5_ levels were also associated with increased outpatient visits [29]. For headache, elevated NO_2_ and PM levels were linked to greater ED visits, with more pronounced effect in winter and spring seasons. The synergistic effect of high temperatures and air pollution increased migraine risk by ~18% [44]. Similarly, high PM_2.5_ and traffic-related pollutants synergistically raised the risk of dysmenorrhea [50]. These findings underscore the substantial pain-related burden associated with high levels of air pollution.

#### 4.2.2. Physiological Vulnerabilities

Women are disproportionately affected by climate change due to physiological factors like pregnancy, heightened by socioeconomic disadvantages and dependency on natural resources [60]. Most studies in this review predominantly included women, consistent with the higher prevalence of pain-related conditions in women [61]. Women are also more likely than men to report weather sensitivity in OA pain and to cite weather changes as migraine triggers [16,47]. Together, these findings emphasize the gendered burden of pain under climate change and the need for effective interventions.

#### 4.2.3. Age

Studies have highlighted that older adults may be more susceptible to climate-related pain. In OA, increased PM_2.5_ exposure was associated with greater pain in older patients [29] Similarly, higher ED visits for headache were linked to dust storms [44]. However, an analysis of 206,789 chest pain cases (mean age: 61.2 years), revealed a stronger correlation between temperature extremes and chest pain in younger individuals, while older adults experienced a more attenuated impact [51].

#### 4.2.4. Socioeconomic Status

Studies indicate that individuals with lower socioeconomic status are more susceptible to temperature-related chest pain [51]. Despite the growing body of literature on climate-related health effects, most studies originated from high-income and upper–middle-income countries, particularly China [62]. Yet climate change disproportionately affects low-income countries (LICs), where it exacerbates pain through heat exposure, extreme weather, environmental degradation, and limited healthcare access [63]. Socioeconomic challenges such as poverty, food insecurity, and displacement further compound the vulnerability, potentially intensifying chronic pain through psychosocial stress and reduced healthcare access [64]. Notably, direct empirical evidence linking climate change to pain outcomes in LICs remains scarce. A global literature analysis found that climate and health research output from high-income countries is nearly 10 times greater than that from LICs [62]. Of the 40 studies reviewed, all except single study conducted in Lebanon originated from high- or upper–middle-income countries, highlighting a significant research gap (Figure 2). This disparity underscores the urgent need for collaborative research initiatives and capacity-building to generate relevant evidence and guide adaptation strategies.

## 5. Research Gaps and Future Perspectives

Climate change significantly impacts pain experiences, disproportionately affecting individuals with chronic pain conditions. Despite this, research at the intersection of climate change and pain remains limited, specifically the studies discussing physiological mechanisms linking climate change and other risk factors to pain [65] Climate change impacts pain through physiological, psychological, and environmental pathways, affecting treatment access and efficacy. Leveraging novel technologies, such as biomarker research on inflammation, oxidative stress, and neurochemical changes, will be crucial for uncovering underlying mechanisms and enabling the development of personalized mitigation strategies [66,67]. At the same time, climate change continues to strain healthcare systems. The healthcare industry itself, including pain management, contributes to carbon emissions, making sustainability a critical component of future mitigation efforts [68]. Models like the Environmental Pain Approach (EPA) advocate for sustainable practice using the ‘Triple R’ framework: Reduce, Replace, and Rethink [69]. Furthermore, targeted interventions and public health campaigns are also essential to protect outdoor workers, who are particularly vulnerable to extreme temperatures. Practical measures such as hydration protocols, shaded workspaces, and adjusted work schedules can help reduce heat-related risks [70]. Similarly, urban planning also plays a crucial role in reducing air pollution [71], which worsens both pain sensitivity and respiratory issues. Expanding green infrastructure, increasing tree coverage, and strengthening air quality regulations at corporate, national, and global levels can improve both public health and workplace safety. Incorporating these measures into pain management and broader public health policies will be critical for building resilience against climate-related health challenges.

## 6. Limitations

Although we performed an extensive literature search, this article is a narrative rather than a systematic review, so the selection of studies may be subject to publication and language bias. We restricted our search to English-language, peer-reviewed articles indexed in PubMed and Embase and did not perform a formal risk-of-bias assessment. As a result, grey literature, conference abstracts, and non-English guidelines may be under-represented. Furthermore, the studies of severe or complex pain conditions such as pain associated with cancer, cardiac discomfort, trigeminal neuralgia, or sickle cell anemia were excluded to preserve interpretability of the environmental exposure-pain relationship. Patients with severe, long-standing pain are frequently subject to intensive, time-varying medical and interventional management (for example, long-term opioids, nerve blocks, or implanted devices), as well as important psychosocial and lifestyle differences. In most primary studies these factors were not measured with sufficient granularity to allow robust control for confounding or effect modification. Including such populations therefore risks producing biased or uninterpretable estimates of the independent contribution of weather or air-quality exposures. Moreover, a study has highlighted that patients with constant severe pain may not notice impact of weather on pain [20], which further limits the detectability of exposure–response relationships in these groups. For these reasons we focused on pain conditions where short-term changes in environmental exposures and symptoms are more readily observable. Because we excluded severe, treatment-intensive chronic pain populations, our findings may not generalize to people with long-standing disabling pain; dedicated longitudinal studies with detailed treatment and medication records are needed to address this gap.

Additionally, considerable heterogeneity in study design and outcome definitions precluded a quantitative meta-analysis; consequently, the recommendations presented here are based on qualitative synthesis and should be interpreted with caution. Moreover, this review may be limited by the absence of negative or null findings, which could reflect either a gap in the literature or the inherent bias of a narrative approach. Unlike systematic reviews, narrative reviews are more prone to selective inclusion, and this should be considered when interpreting the results. Like, four studies analyzed the effect of weather parameters on chronic pain using UK-based smartphone app, which may pose various limitations such as selection bias, low participant engagement and recruitment spikes may lead to biasness of results, effect on data quality due to inconsistency in pain scores used making comparison difficult, and limiting generalizability due to region-specific survey [20,21]. Additionally, the broad discussion of different pain types may lack depth in explaining their distinct mechanisms in relation to environmental factors. A single overarching conclusion might oversimplify the complexity of pain experiences across diverse populations, thereby reducing generalizability. Additionally, the inclusion of both climate change and air pollution each influenced by weather variability and anthropogenic activities makes it challenging to disentangle their individual contribution to pain outcomes. Finally, neglecting confounding variables like extreme weather’s impact on mood, sleep, or healthcare access may limit the accuracy and comprehensiveness of this review.

## 7. Conclusions

Recognizing the connection between weather patterns and pain could enhance diagnostic accuracy and inform more effective treatment strategies. Chronic conditions like OA, dysmenorrhea, and gout are primarily influenced by humidity, temperature, and barometric pressure, with symptoms often worsening in colder or more humid conditions. In contrast, acute pain conditions such as headaches and postoperative pain show stronger links with temperature extremes, humidity fluctuations, and air pollution.

The most consistently observed associations include greater pain with higher humidity/dampness in patients with knee OA, an increased risk of headache/migraine with higher humidity/rainfall, and increased dysmenorrhea pain with colder temperatures. Headache was often reported following exposure to extreme heat. Higher levels of air pollutants may be associated with increased risk of migraine and dysmenorrhea as well as higher rates of medical visits for knee OA and headache. Recognizing environmental triggers of pain allows for more precise and targeted management strategies, advancing climate-conscious healthcare solutions. Adaptation strategies should emphasize climate-responsive healthcare systems, pollution control, personalized pain management, biomarker integration, and public awareness to address pain risks linked to climate change and air pollution. Further research is warranted to evaluate the impact of these exposures in vulnerable populations, particularly women and individuals in low- and lower–middle-income countries. Strengthening the scientific understanding in these areas can inform policy development and promote more equitable healthcare interventions.

## Figures and Tables

**Table 1:** Relationship between weather parameters and chronic pain across multiple conditions: Findings using a UK-based smartphone application (“Cloudy with a Chance of Pain”).

Study	Study Design and Participants	Key Findings
Patel et al., 2016 [18]	Analysis of data from 5782 respondents aged ≥ 17 years with chronic pain (71% female)	Median (IQR) belief strength (weather associated with pain) was 7 (5–9) ^a^ Participants most commonly endorsed that “damp/rainy” season affected their symptoms (72%) Compared with the total study population, pts with FM, chronic headache, and neuropathic pain more frequently reported that changes in temperature and pressure correlated with pain Relative to the total study population, pts with FM and neuropathic pain more commonly believed that all aspects of weather (temperature change, damp/rainy, pressure change, hot, and cold) were associated with pain Similar percentages of men and women reported associations between weather aspects and pain Compared with those aged < 50 years, individuals ≥ 50 years were less likely to report an associated with temperature change (22% vs. 34%) and cold (61% vs. 72%)
Dixon et al., 2019 [19]	Multivariate case-crossover analysis of weather variables; included 2658 adults aged ≥ 17 years (83% female; mean [SD] age, 51 [12.6] years) with chronic pain (>3 months; including unspecified arthritis, OA, gout, RA, chronic headache/migraine, neuropathic pain, FM/chronic widespread pain, and other/no medical diagnosis); participants were followed for up to 15 months and scored days as either having no pain events or having pain events	Higher relative humidity and wind speed and lower atmospheric pressure were significantly associated with increase pain severity, with relative humidity showing the largest effect Odds of a pain event increase by 4.5% for every 5 mph increase in wind speed and 13.9% for every 10% increase in relative humidity, and decrease by 3.8% for every 10-mbar increase in pressure Temperature was not significantly associated with pain In analyses by pain state, relative humidity was significantly associated with osteoarthritis pain, but results for other conditions inconclusive due to limited statistical power
Schultz et al., 2020 [20]	Analysis of data from 10,584 participants ^b^ (81% female; mean [SD] age, 48 [13.2] years [11]) with chronic pain (including unspecified arthritis, OA, gout, RA, chronic headache/migraine, neuropathic pain, FM/chronic widespread pain, and other/no medical diagnosis) examining the association between pain events (days with pain) at the population level and weather patterns (climate and atmospheric conditions)	Median (IQR) belief strength (weather associated with pain) was 8 (6–9) ^a^ A high percentage of participants experienced pain events on days (top 10% of days [45 days] with largest percentage of participants having a pain event) with lower BMP Low BMP was generally associated with more wind, moisture, and precipitation Days when a low percentage of participants experienced pain events (bottom 10% of days [45 days] with lowest percentage of participants having a pain event) were characterized by higher BMP, which was associated with weaker winds and dryer air
Yimer et al., 2022 [21]	Bayesian multilevel model examined association between weather and pain; analysis included 6213 pts (82.4% female; mean [SD] age, 48.7 [13.0] years) with chronic pain conditions (including unspecified arthritis, OA, gout, RA, chronic headache/migraine, neuropathic pain, FM/chronic widespread pain, and other/no medical diagnosis); correlated pain data with objective weather data	Median (IQR) belief strength (weather associated with pain) was 7 (6–9) Higher relative humidity, higher wind speed, lower temperatures, and lower BMP associated with increase pain, but associations were modest After adjusting for age, sex, belief in weather–pain association, mood, and activity level, 1 in 10 pts were sensitive to temperature, 1 in 25 to relative humidity, 1 in 50 to BMP, and 3 in 100 to wind speed; directionality (i.e., higher or lower values associated with pain) was variable Findings regarding sensitivity largely consistent across conditions

^a^ Questionnaire assessed belief strength (how likely do you think weather is associated with pain?) on a scale from 0–10 and belief type (if you think there is a possible association, which weather condition [s] affect your pain the most?). ^b^ This was the same cohort that had complete baseline information and at least 1 pain entry in the studies [19,21]; the analysis presented in Dixon et al. [19] was confined to the 2658 participants from this cohort that had at least 1 hazard period (with pain) matched to a control period (without pain) in the same month, while the analysis by Yimer et al. [21] included 6213 participants who had completed baseline information, had ≥2 days of pain reports, and had hourly location data sufficient to access complete weather information in order to calculate daily means. BMP, barometric pressure; FM, fibromyalgia; IQR, interquartile interval; OA, osteoarthritis; pts, participants; RA, rheumatoid arthritis; SD, standard deviation.

## Data Availability

No new data was created or analyzed in this study. Data sharing is not applicable to this article.

## Abbreviations

The following abbreviations are used in this manuscript:

BMP	Barometric pressure
CGRP	Calcitonin gene-related peptide
CI	Confidence interval
CO	Carbon monoxide
ED	Emergency department
EPA	Environmental pain approach
EPOSA	European Project on Osteoarthritis
LBP	Low-back pain
LIC	Low-income countries
NO	Nitric oxide
NO_2_	Nitrogen dioxide
NOx	Nitric oxides
O_3_	Ozone
OA	Osteoarthritis
OR	Odds ratio
PM	Particulate matter
PR	Prevalence ratios
PROs	Patient-reported outcomes
QoL	Quality of Life
RA	Rheumatoid arthritis
RR	Relative risk
SD	Standard deviation
TRP	Transient receptor potential
TTH	Tension-type headache
